# Development and Characterization of Thermoresponsive Smart Self-Adaptive Chitosan-Based Polymer for Wellbore Plugging

**DOI:** 10.3390/polym15244632

**Published:** 2023-12-07

**Authors:** Huimei Wu, Yishan Lou, Zhonghui Li, Xiaopeng Zhai, Fei Gao

**Affiliations:** 1 National Engineering Research Center for Oil & Gas Drilling and Completion Technology, Yangtze University, Wuhan 430100, China; louys2006@126.com (Y.L.);; 2 Hubei Key Laboratory of Oil and Gas Drilling and Production Engineering, Yangtze University, Wuhan 430100, China

**Keywords:** temperature-switchable, intelligent fluid, adaptive, rheological properties, filtration loss

## Abstract

To meet the escalating demand for oil and gas exploration in microporous reservoirs, it has become increasingly crucial to develop high-performance plugging materials. Through free radical grafting polymerization technology, a carboxymethyl chitosan grafted poly (oligoethylene glycol) methyl ether methyl methacrylate acrylic acid copolymer (CCMMA) was successfully synthesized. The resulting CCMMA exhibited thermoresponsive self-assembling behavior. When the temperature was above its lower critical solution temperature (LCST), the nanomicelles began to aggregate, forming mesoporous aggregated structures. Additionally, the electrostatic repulsion of AA chains increased the value of LCST. By precisely adjusting the content of AA, the LCST of CCMMA could be raised from 84.7 to 122.9 °C. The rheology and filtration experiments revealed that when the temperature surpassed the switching point, CCMMA exhibited a noteworthy plugging effect on low-permeability cores. Furthermore, it could be partially released as the temperature decreased, exhibiting temperature-switchable and self-adaptive plugging properties. Meanwhile, CCMMA aggregates retained their reversibility, along with thermal thickening behavior in the pores. However, more detailed experiments and analysis are needed to validate these claims, such as a comprehensive study of the CCMMA copolymer’s physical properties, its interaction with the reservoir environment, and its performance under various conditions. Additionally, further studies are required to optimize its synthesis process and improve its efficiency as a plugging material for oil and gas recovery in microporous reservoirs.

## 1. Introduction

As conventional oil and gas fields become depleted, unconventional fields like shale oil and tight oil have gained increasing attention. These fields are often characterized by a high concentration of clay and fine pores, posing unique challenges for geological conditions [1,2]. This can result in formation damage and wellbore instability during the drilling process, primarily due to fluid leakage, which can lead to contamination near the wellbore. Therefore, the development of high-performance plugging agents is crucial for ensuring the smooth operation of drilling projects in these challenging geological conditions.

Currently, a range of materials are available for use as plugging agents, offering a higher level of adaptability for plugging nanoscale pores compared to traditional agents, which often consist of rigid particles and composite materials [3,4]. While inorganic nanoparticles can indeed reduce the permeability of low-permeability reservoirs to some extent, their plugging effectiveness is highly dependent on the relative relationship between the nanoparticles and the pores [2,5]. Therefore, it is essential to consider the pore size of the formation when selecting the appropriate size of plugging agents during the plugging process. Inorganic plugging agents, although effective, can be outperformed by polymer nanoparticles, polymer gels, and corresponding inorganic–organic composite materials due to their inherent deformability, which facilitates the formation of tight seals [6,7,8,9,10,11,12]. These polymer-based plugging materials possess good colloidal stability, can be dispersed in water without agglomeration, and can be readily customized to incorporate various functions. This provides them with a distinct advantage over inorganic plugging agents.

In order to improve the effectiveness of organic polymer sealing materials, domestic and foreign scholars have carried out a series of developments of temperature-sensitive intelligent polymers. Common temperature-sensitive polymers include polyacrylamide, polyethylene ether, and polymethyl methacrylate. Poly (N-isopropylacrylamide) (PNIPAM) polymer is currently the most researched and technologically mature thermosensitive polymer. Lei et al. [13,14] proposed a self-crosslinking nanoplugging agent that can react within a certain temperature range to form excellent plugs in micropores [15,16]. With the study of plugging agents, temperature-responsive polymers are increasingly gaining attention due to their efficient sealing properties. Materials such as poly(N-isopropylacrylamide) (PNI) and N-vinylcaprolactam (NVCL) side-chain cross-linked polymers all exhibit thermally thickening behavior in plugging operations due to their special thermal binding properties [17,18]. Du et al. [19] synthetized a β-cyclodextrin gel that can undergo sol–gel–sol transition when temperature changes, which can be used as a temporary plugging agent for cracks. Dong et al. [20] synthesized a bentonite containing intermeshed PNI, which has good sealing effect, good economy, and thermal response characteristics. Bai et al. [21] developed a temperature-responsive plugging agent composed of NIPAM-grafted silica nanoparticles. These studies demonstrate the effectiveness of temperature-responsive plugging agents [22]. Lei et al. [23] grafted MAOEM with carboxymethyl chitosan (CMCS) to preliminarily prepare an environmentally friendly temperature-responsive polymer, which can reduce the filtration loss of drilling fluids and inhibit clay swelling properties.

However, existing thermosensitive polymer sealing agents still face the challenge of low usage temperatures, with the LCST of conventional thermosensitive polymers being less than 50 °C. Additionally, their compatibility in drilling fluid is poor, and they tend to agglomerate with clay particles, resulting in poor dispersion stability, which significantly impacts the rheological properties of the drilling fluid. To address the issue of drilling fluid leakage under complex geological conditions and to overcome the limitations of traditional sealing materials in terms of sealing efficiency, environmental friendliness, and temperature resistance, an innovative and reusable sealing material has been developed. The focus of this study was on the directional plugging of microporous formations. To achieve this, CCMMA was prepared by grafting AA and MAOEM onto a CMCS skeleton with different mole ratios.

## 2. Materials and Methods

### 2.1. Experimental Materials

Chitosan (low viscosity, less than 200 mPa·s) and MAOEM (Mn~300 g/mol) were procured from Aladdin Reagent Company (Shanghai, China). Isopropanol (AR, ≥99.7%), ethanol (AR, water ≤ 0.3%), NaOH (AR, 96%), chloroacetic acid (99%), and APS (AR, ≥98%) were purchased from Chengdu Kelong Chemical Reagent Factory (Sichuan, China). All of the raw materials mentioned above are of analytical grade and require no further purification. The deionized water used in the experiments was synthesized in the laboratory.

### 2.2. Preparation of Chitosan-Based Polymers

**Figure 1:**
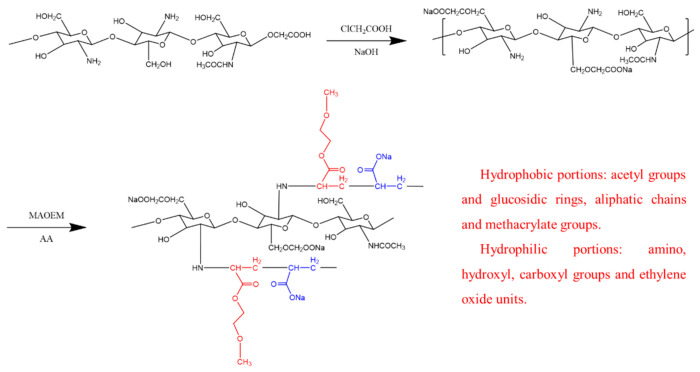
Graft modification route of CCMMA. Different colors show the grafting of AA and MAOEM.

In this study, chitosan carboxylation and grafting were conducted to obtain CMCS for the subsequent plugging work. Specifically, the experimental modification and grafting work can be divided into the following steps:

1. To dissolve the chitosan, we placed 20 g of it into a conical flask. Remembering that chitosan is soluble in acidic solutions, we added 32 g of NaOH as a solvent. This alkaline environment not only ensures sufficient carboxymethylation modification of chitosan but also reduces raw material consumption. After vigorously mixing the contents in an ice-water bath for 30 min, we obtained a well-mixed chitosan suspension.
2. Chitosan modification: Due to the nucleophilic reaction between the hydroxymethyl groups (-CH_2_OH) on chitosan and the amide groups (-NH_2_), chitosan, a biopolymer, possesses multiple hydroxymethyl sites. Gel-like polymer structures can be achieved through crosslinking reactions. To modify the chitosan, 30 g of chloroacetic acid was added as an etherifying agent, followed by 50 milliliters of isopropanol as a solvent to the chitosan suspension. The chitosan suspension was then placed in a 50 °C environment for 10 min. The modification process for chitosan is depicted in Figure 1. Subsequently, 500 milliliters of a 70% ethanol solution was added to the mixture and stirred at room temperature for one hour. The solid material was then filtered out of the suspension.
3. Extraction of CMCS: The solid particles were rinsed in a 75% ethanol solution to remove any precipitated sodium salts and any residual water. The product was then thoroughly dried under vacuum to obtain preliminarily purified biomass CMCS. To obtain completely water-soluble CMCS, the water extraction method was employed. For this, the crude salt product was mixed with deionized water in a ratio of 1:30. Then, a 1.3 mol/L HCl neutralization solution was used to remove insoluble impurities (alkali salts) by precipitation. After collecting the solid particles, purified CMCS was obtained through vacuum drying, with a yield of approximately 94%.
4. Preparation of CMCS: To prepare CMCS, the material was dissolved in deionized water and nitrogen gas was continuously bubbled through the solution. The solution was then heated to 70 °C for 2 h. After this, 0.2 g of ammonium persulfate (APS) was added as an initiator and the mixture was stirred for 20 min to prevent the aggregation effect of the product.
5. Free radical grafting of CMCS: Different ratios of solutions of MAOEM and AA were mixed and added to the CMCS solution. To enhance the grafting reaction, nitrogen gas was bubbled through the mixture while maintaining it at 70 °C. To account for the varying proportions of MAOEM in the solvent system, experimental groups were designated as CCMMA-A to CCMMA-F, as shown in Table 1. The grafting process is schematically represented in Figure 1.
6. Purification of CCMMA: The grafted CCMMA polymer was completely immersed in deionized water. The deionized water was replaced every 5 h to remove unreacted CMCS and chitosan monomers. After purification, the product was dried in a vacuum oven at 60 °C for 3 h to obtain purified CCMMA [24].

### 2.3. Characterization of Chitosan-Based Polymers

The calculation equations for the grafting rate and grafting efficiency of CCMMA are as follows [25]:

(1) $$GR=\frac{m_{CCMMA}-m_{CMCS}}{m_{CMCS}}\times 100\%$$

(2) $$GE=\frac{m_{CCMMA}-m_{CMCS}}{m_{monomers}}\times 100\%$$

where *m_C_*, *m_G_*, and *m_M_* are the masses of CMCS, COMA, and total monomers, respectively.

#### 2.3.1. Microstructure Characterization

The structure of CCMMA was analyzed using a Nicolet 6700 FT-IR spectrometer (Beijing Youran Ruizhi System Technology Co., Ltd., Beijing, China) and ^1^H NMR spectroscopy. The FT-IR spectra were recorded in the range of 450 to 4000 cm^−1^, while the ^1^H NMR spectrum was acquired in D_2_O at a temperature of 20 °C.

#### 2.3.2. Self-Assembly Behavior Testing

The hydrodynamic diameter (D_h_) of CCMMA was measured using a nanoparticle size analyzer (Brookhaven, Holtsville, NY, USA), and particle size distribution was utilized to examine the self-assembly behavior of CCMMA.

#### 2.3.3. Turbidity Testing

Turbidity measurements and DLS were employed to investigate the temperature-responsive behavior of various CCMMA samples. The turbidity of the CCMMA solution was measured at different temperatures using an OUM 223/253 turbidimeter (AMER, Anaheim, CA, USA) with a water bath. Additionally, an extrapolation was performed to estimate the cloud point above 100 °C [26]. DLS measurements were conducted to compare the particle sizes of CCMMA below and above the LCST.

#### 2.3.4. Drilling Fluid Performance Testing

(1) Rheological and Filtrate Loss Testing: To investigate the influence of CCMMA on sodium-based bentonite (Na-B) dispersions, rheological and filtrate loss experiments were conducted. For the preparation of Na-B dispersion, 0.75 g of Na_2_CO_3_ and 20 g of Na-B were added to 600 mL of water. The mixture was stirred vigorously for 1 h and then sealed for 36 h. Different concentrations of CCMMA were then introduced into the solidified Na-B dispersion, resulting in the formation of Na-B/CCMMA dispersions. The rheological characteristics of the Na-B/CCMMA dispersions were evaluated both before and after hot rolling at 130 °C. The temperature and rheological parameters were measured using a ZNN-D6 rotational viscometer (Shanghai Shengshi Huike Testing Equipment Co., Ltd., Shanghai, China) and calculated by applying Equations (3)–(5).

(3) $$YP=\frac{2\times \emptyset 300-\emptyset 600}{2}$$

(4) PV=∅600−∅300

(5) $$AV=\frac{\emptyset 600}{2}$$

where ∅400 and ∅200 represent the readings of the viscometer.

To gain a deeper understanding of the fundamental interaction mechanisms between clay polymer and dispersed bentonite particles, filtration tests were conducted using a custom-built filtration device under a pressure of 0.70 MPa for one hour. Additionally, the particle size distribution (PSD) of both Na-B and Na-B/CCMMA dispersions was analyzed using a laser particle size analyzer. This analysis aimed to further elucidate the effects of CCMMA on the behavior of dispersed bentonite particles.

(2) High-temperature, high-pressure rheological and filtrate loss testing: Temperature sweep rheological tests were performed using an ARES-G2 rotational rheometer. The filtration loss testing involved two stages: (1) increasing the temperature from 20 °C to 130 °C (2 MPa) and (2) maintaining the temperature at 130 °C while gradually increasing the pressure difference by 1 MPa every 5 min until it reached 6 MPa.

To gain insight into the interaction between Na-B and CCMMA at varying temperatures, we carried out isothermal adsorption experiments. In these experiments, 0.5 g of dry Na-B was introduced into CCMMA solutions with concentrations ranging from 100 to 2000 mg/L. The mixtures were then shaken for 36 h at both 20 °C and 90 °C. After the shaking process, the suspensions were centrifuged, and both the supernatant and the original solution were collected. These samples were then appropriately diluted and analyzed using a TOC-V analyzer (Shimadzu, Kawasaki, Japan) to determine both the equilibrium and initial concentrations. The difference between these two values enabled us to calculate the equilibrium adsorption capacity.

#### 2.3.5. Formation Plugging Performance Testing

**Figure 2:**
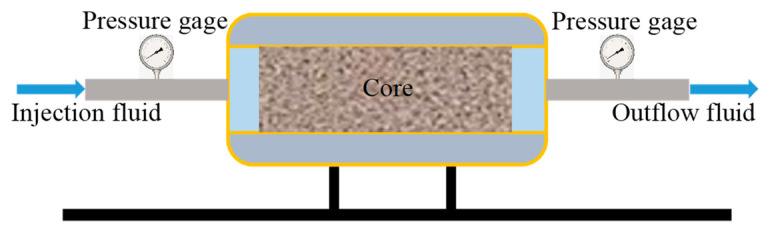
Schematic of rock pressure transmission experiment equipment.

A core pressure transmission test was conducted to measure the stable pressure at both the upstream and downstream locations. The rock permeability was then calculated using the Darcy equation, incorporating the experimental data [27,28,29,30,31,32,33]. For the experimental setup, the upstream pressure was set at 3 MPa, the downstream pressure at 1 MPa, and the confining pressure at 6 MPa. A 3% NaCl solution was used for testing as a comparison with PNI, which is one of the most mature temperature-responsive polymers. The performance of different plugging agents in the core throats was evaluated by calculating the core permeability after plugging. Refer to Figure 2 for a schematic diagram of the pressure transmission test apparatus.

## 3. Results and Discussion

### 3.1. Microstructure Characterization

#### 3.1.1. FT-IR Spectrum Analysis

**Figure 3:**
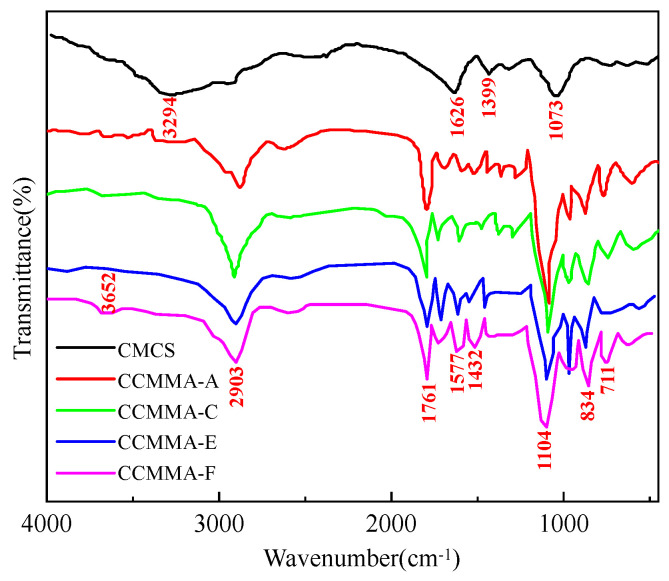
FT-IR spectra of CCMMA and CMCS.

To analyze the structure of CCMMA upon the addition of MAOEM and AA, Figure 3 displays the results of the FT-IR spectrum analysis.

The FT-IR spectrum of CCMMA displays additional absorption peaks, including a peak at 1761 cm^−1^ due to the C=O group in MAOEM and a peak at 1104 cm^−1^ assigned to the C-O-C group of the ethoxy group [34]. Furthermore, the band at 2903 cm^−1^ in the CCMMA sample indicates an increase in the stretching vibration of aliphatic C-H bonds, while the peak related to N-H stretching vibration shows a decrease. The differences in stretching vibrations between CCMMA and CMCS suggest the successful grafting of MAOEM onto the main chain, primarily targeting the amino groups. With higher AA content among different CCMMA samples, the intensity of the -COO- vibration band enhances. The -COO- vibration peak also shifts to lower wavenumbers, and the peak associated with N-H vibrations shifts to lower frequencies, suggesting the formation of more hydrogen bonds between -OH/NH_2_ and -COO groups [35]. Therefore, the successful modification of CCMMA has been verified.

#### 3.1.2. ^1^H NMR Analysis

**Figure 4:**
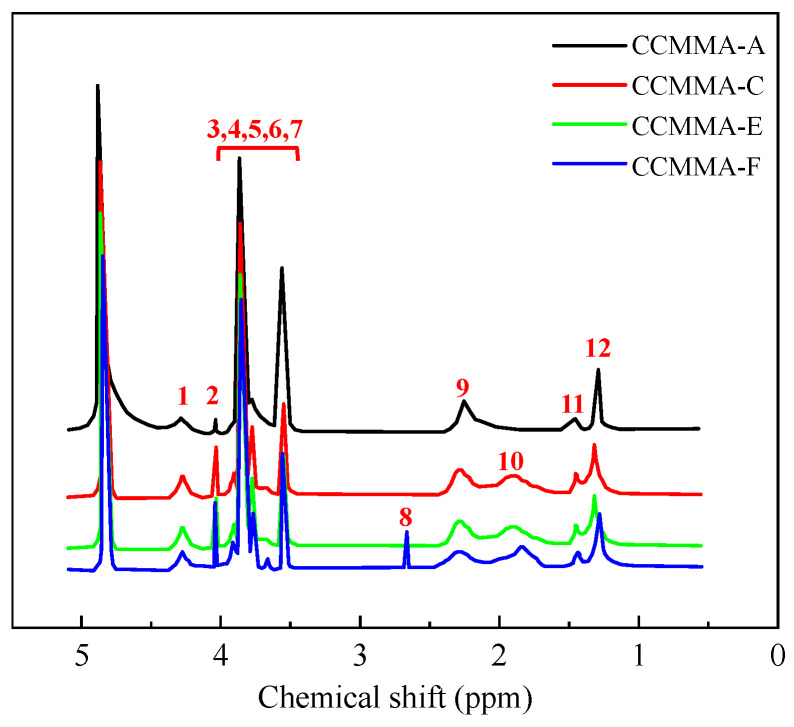
^1^H Nuclear Magnetic Resonance spectrum (D_2_O, 20 °C).

To further investigate the impact of the AA content on the synthesis process, Figure 4 presents the results of the ^1^H NMR analysis. This figure reveals that all three CCMMA samples exhibit comparable molecular structures, as indicated by their similar resonance peaks [36,37,38].

Signals at 1.23–1.49 ppm and 2.60–2.68 ppm represent -C-CH_3_ and -COCH_3_ groups, respectively. The multi-peaked signal between 2.40 and 2.44 ppm is attributed to -CH- protons. The signal at 1.73–1.84 ppm corresponds to the -CH_2_- protons in PMAOEM and PAA chains. Signals ranging from 3.46 to 3.96 ppm are attributed to the pyran ring and carboxymethyl groups.

Furthermore, the intensity of proton signals of AA units varies significantly. As the molar ratio of AA feed increases (no resonance signal observed for CCMMA-A), the FT-IR results indicate that more AA segments were incorporated into the polymer structure. Additionally, an increase in the AA content causes a shift of the acetylaminomethyl proton signal in CMCS to higher fields, suggesting that the introduced carboxylic acid groups prevent the formation of hydrogen bonds [39,40]. This interplay maintains the stretching of CMCS chains, facilitating grafting reactions. The calculated molar ratios of MAOEM and AA in CCMMA, based on Equation (6), are as follows:

(6) $$\frac{n_{O}}{n_{A}}=\frac{S_{1}}{3\times S_{2}}$$

In the equation, *n_O_* and *n_A_* represent the amounts of MAOEM and AA, respectively, in moles. *S*_1_ represents the integration of -C-CH_3_ in the MAOEM, while *S*_2_ represents the integration of -CH_2_- in the AA. As shown in Table 1, the calculated mole ratio values (CCMMA-A 10/0, CCMMA-D 7.1/2.9, and CCMMA-F 5.3/4.7) closely match their respective feed mole ratios. This confirms that the MAOEM and AA monomers undergo nearly complete conversion during the grafting process. The higher content of MAOEM in the synthesized graft copolymer compared to the feed mole ratio is due to the higher reactivity of MAOEM, as discussed earlier.

### 3.2. Analysis of Self-Assembly Behavior

**Figure 5:**
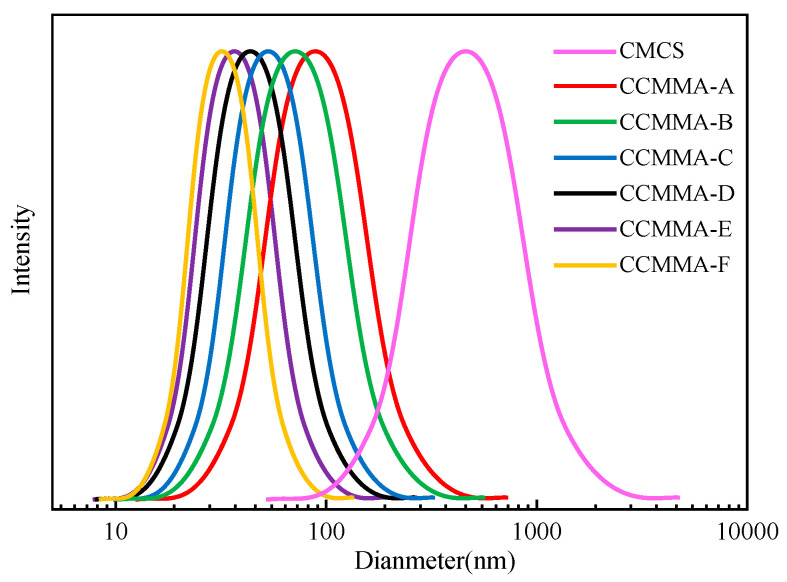
Size distribution of CMCS and CCMMA as measured by DLS (20 °C).

As shown in Figure 5, the average Dh of the original CMCS was relatively high at 534.13 ± 15.76 nm. The fluid dynamic volume of CMCS is determined by the aggregation structure driven by hydrophobic interactions and electrostatic forces between molecules. The grafting of AA units onto the MAOEM backbone leads to a reduction in fluid dynamic volume, with greater incorporation of AA resulting in smaller values of Dh. This prevents the formation of large aggregates by the grafted (MAOEM-co-AA) chains, especially for copolymers with higher AA content. These changes in molecular structure weaken intermolecular hydrogen bonding, increase interchain steric hindrance, and enhance interchain electrostatic repulsion. This impedes the aggregation between CCMMA molecules, while promoting the self-assembly of CCMMA into compact nanostructures. The increased poly(acrylic acid) (PAA) segments in CCMMA-F increase negative charge density, leading to stronger electrostatic repulsion and limiting polymer aggregation. DLS analysis confirms the self-assembly behavior of the synthesized CCMMA copolymer in aqueous solution, which is influenced by the molar composition of MAOEM/AA.

**Figure 6:**
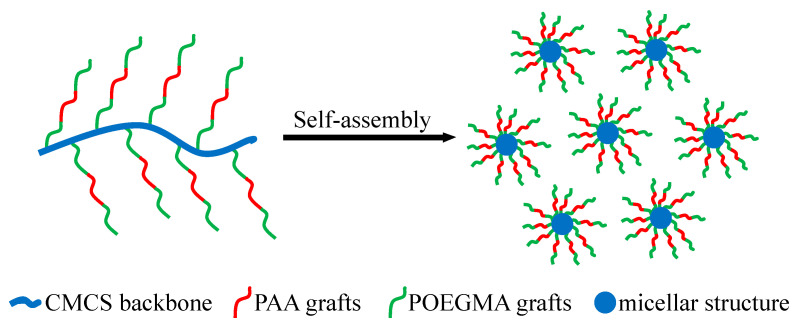
Schematic representation of the self-assembly mechanism.

Given the amphiphilic structure of CCMMA, which contains both hydrophobic and hydrophilic portions, its self-assembly behavior is illustrated in Figure 6. This amphiphilic structure promotes the formation of micelles through interactions between the hydrophobic segments and hydrogen bonding between the hydrophilic components [41,42]. These structures lead to the formation of compact micellar structures, with the hydrophilic shell formed by the polymer grafts (MAOEM-co-AA) surrounding the framework.

### 3.3. Analysis of Temperature-Responsive Behavior

**Figure 7:**
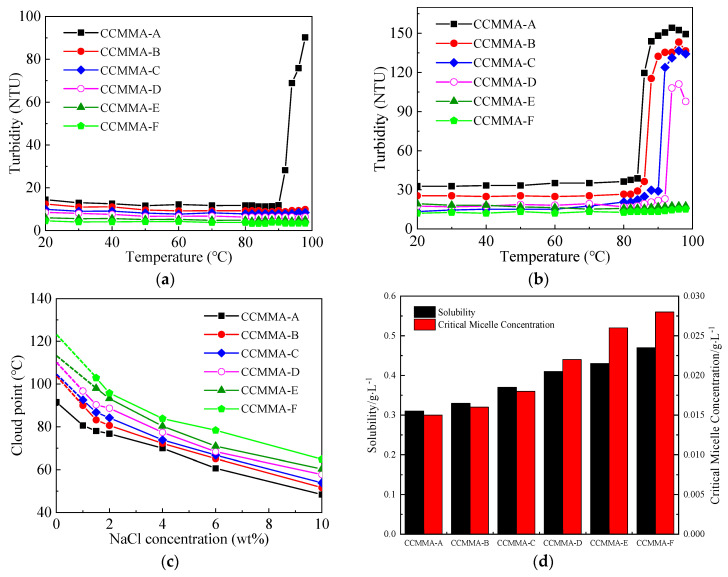
Temperature-responsive behavior of the polymer; (**a**) turbidity–temperature curve (2 wt%); (**b**) turbidity–temperature curve (4 wt%); (**c**) turbidity–NaCl concentration curve; (**d**) solubility and CMC of CCMMA.

As shown in Figure 7a, for 2 wt% CCMMA solutions, CCMMA-A displayed a cloud point of 90.3 °C. However, when the molar content of AA exceeded 10%, CCMMA maintained a low turbidity level, even at 100 °C, indicating no phase separation. When the polymer concentration was increased to 4 wt%, stronger polymer–polymer interactions resulted in a decrease in the cloud point (Figure 7b). However, no cloud points were observed for CCMMA-E and CCMMA-F, suggesting that their transition temperatures in this concentration range were higher than 100 °C. Therefore, the LCST behavior of CCMMA is induced by the MAOEM component due to hydrogen interactions and hydrophobic effects within the polymer [43]. The introduction of hydrophilic AA chains has a significant impact on this LCST behavior. As the content of AA chains increases in the CCMMA structure, the LCST value also increases. This can be attributed to improved polymer–water interactions resulting from increased hydrophilicity. Nevertheless, when temperatures exceed the cloud point, a higher AA content leads to decreased turbidity, indicating reduced polymer–polymer interactions during phase separation and a diminished likelihood of CCMMA chain aggregation.

In Figure 7c, it can be observed that as the NaCl concentration increased, the cloud points of CCMMA samples decreased. NaCl triggered partial dehydration of the CCMMA chains by capturing water molecules surrounding the polymer, thereby reducing the polymer–water interactions [44]. Additionally, the shielding effect between the charged carboxyl groups on the main chain and side chains promoted the interaction between polymers, leading to easier sedimentation and a lower cloud point. The effect was more pronounced with higher NaCl concentration or ion strength. However, different decreasing trends were observed for CCMMA samples with and without AA (acrylic acid) chains. For CCMMA-A, as the NaCl concentration increased to 10 wt%, the cloud point decreased to 50.7 °C. CCMMA-A graft copolymers typically exhibited LCST behavior similar to reported PMAOEM homopolymers [45], showing a linear relationship between cloud point and ion concentration in PEG systems. Therefore, LCST behavior is primarily influenced by the PMAOEM graft chains and is not strongly correlated with the main chain. However, the addition of AA increased the sensitivity of CCMMA to salt, especially when the salt concentration was below 2 wt%.

Figure 7c reveals that as the NaCl concentration increased, the cloud points of CCMMA samples exhibiting LCST shifted significantly downward compared to CCMMA-A. This decrease became less severe as the salt concentration surpassed 2 wt%. These results suggest that there is almost a linear correlation between LCST and the molar ratio of AA. Additionally, Figure 7d demonstrates that as the AA content increases, the solubility and CMC of CCMMA are effectively enhanced due to the addition of hydrophilic groups. This observation aligns with the conclusions drawn from turbidity testing.

**Figure 8:**
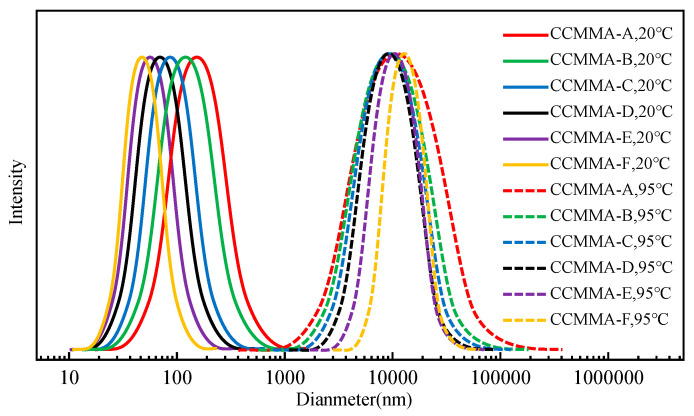
DLS size distribution of different CCMMA samples in a 2 wt% NaCl solution.

**Figure 9:**
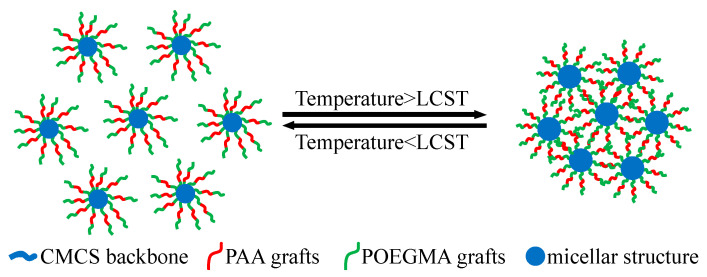
Schematic diagram of thermally induced self-assembly.

To ensure that the phase transition temperature remained below 100 °C, a concentration of 5 wt% NaCl was added to the samples. As shown in Figure 8, the addition of NaCl led to an increase in D_h_ value, further indicating that the screening effect can promote molecular chain aggregation. When the temperature exceeded the LCST, which was accompanied by macroscopic phase separation, the D_h_ values of all CCMMA samples suddenly increased to above 8000 nm. At this point, CCMMA formed spherical nanoassemblies with a hydrophilic graft shell and a core composed of interconnected hydrophobic CMCS (core–multishell) chains formed by intermolecular hydrogen bonding. Additionally, above the LCST, the dehydration of graft chains would result in chain contraction and collapse of the hydrophobic domains, leading to a more compact structure with a lower fluid dynamic volume [46]. The sharp increase in D_h_ values signified the preponderant hydrophobic interactions between the pendant graft chains on the surface of the neighboring nanocomponents, fostering the secondary aggregation of micelles. As Figure 9 reveals, this validated the proposed self-assembly structure of CCMMA. Consequently, above the LCST, individual nanocomponents transitioned into aggregated microcomponents. This endows CCMMA with the captivating potential to exhibit temperature-responsive intelligent blocking behavior in mesoporous media, an avenue that will be further explored in future investigations.

### 3.4. Evaluation of Drilling Fluid Performance

#### 3.4.1. Rheological and Filtration Evaluation

**Figure 10:**
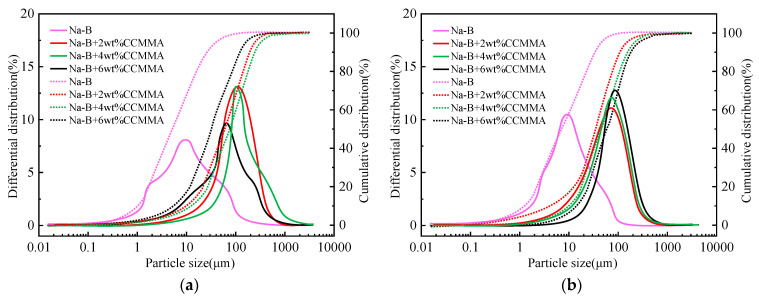
Particle size distribution (solid line: differential distribution; dashed line: cumulative distribution). (**a**) Before hot rolling; (**b**) after hot rolling.

Table 2 presents the impact of CCMMA on Na-B dispersions. As shown in Figure 10, an increase in CCMMA concentration led to a rise in viscosity and a decrease in filtration loss. While CCMMA had a minimal effect on the strength of bentonite particles, the PMAOEM side chains established hydrogen bonds between the ethoxy groups and hydroxyl groups in the clay particles, leading to bentonite aggregation and a reduction in strength. Conversely, interparticle cohesion through CCMMA chains enhanced the structure. These two effects canceled each other out, resulting in almost no change in the yield point. Notably, the introduction of CCMMA caused a significant increase in the median particle size of bentonite particles (see Figure 10a), confirming bentonite aggregation. This aggregation created larger particles within the filter cake, ultimately leading to increased filtration loss in the Na-B dispersion system. Therefore, CCMMA regulated the filtration capability mainly by enhancing the viscosity of the solution, impeding water flow, and forming a polymer film that prohibited water intrusion, mitigating the detrimental effects of aggregation. Additionally, as the CCMMA content rose, the carboxyl groups increased, partially disrupting particle aggregation through electrostatic repulsion. Overall, when the CCMMA concentration was high, the rheological and filtration properties of the Na-B dispersion system were influenced by viscosity, particle aggregation, and the formation of a polymer film.

The high temperature promotes the dispersion of particles and facilitates interparticle bonding, resulting in increased strength [47]. The formation of weak flocculated structures also contributes to an increase in filtrate volume. However, when the hot rolling temperature is 130 °C, the viscosity and yield point of the Na-B dispersion experience a significant decrease. This is due to aging-induced aggregation (surface bonding) and the increase in D50 value after aging (see Figure 10b). With an increase in hot rolling temperature, the viscosity of Na-B/CCMMA dispersion decreases, while the yield point remains unchanged. Furthermore, the filtrate volume of Na-B/CCMMA dispersion shows a slight increase, but it consistently remains lower compared to that of Na-B dispersion. However, the filtrate volume significantly increases after hot rolling at 130 °C. This may be due to the degradation of CCMMA at high temperatures, weakened film-forming effect, and difficulty in balancing the adverse effects of aggregation on liquid loss.

The D50 value of Na-B/CCMMA suggests that there is a partial loss of interaction between degraded CCMMA and bentonite. However, the D50 value of Na-B/CCMMA dispersion after 2 wt% and 4 wt% aging is lower, indicating that the changes in the hydrophilicity of CCMMA do not affect the re-dispersion of the Na-B dispersion system. Furthermore, the original CMCS has diluting and reducing filtrate characteristics [48]. Based on a comparison of the rheological and filtrate properties of CCMMA and CMCS in a Na-B dispersion system, it can be concluded that CCMMA exhibits superior thickening effects while retaining the structural integrity of bentonite particles. This can be attributed to the loss of amphoteric properties in CCMMA following the consumption of amino groups. As a result, the hydrated Na-B aggregates through the PMAOEM side chains rather than being dissociated by the amphoteric structure of CMCS. However, the aggregation of Na-B/CCMMA induced by the PMAOEM grafting agent leads to a wider range of water loss, particularly after thermal aging. This is because the thermohydrolyzed CMCS can still disintegrate and retain finer bentonite particles, thereby reducing filtrate loss. Hence, the introduction of the PMAOEM grafting agent yields a different effect of CCMMA on the dispersion of Na-B compared to its precursor, with the aggregation of bentonite particles by the PMAOEM grafting agent being the primary factor. Additionally, the yield point of Na-B/CCMMA decreases after aging at 100 °C and 115 °C as compared to the Na-B dispersion, indicating that CCMMA can suppress the hydration dispersion of bentonite particles at high temperatures. Further investigation and analysis will be conducted in the subsequent inhibition evaluation section.

The findings indicate that the incorporation of CCMMA into a Na-B dispersion leads to the formation of a polymer film via the aggregation of bentonite particles. This results in increased viscosity and reduced filtrate volume, with the formation of the polymer film contingent on the concentration of CCMMA. Moreover, the impact of CCMMA on Na-B dispersion is retained even after hot treatment at 130 °C.

#### 3.4.2. High-Temperature Rheological and Filtration Evaluation

**Figure 11:**
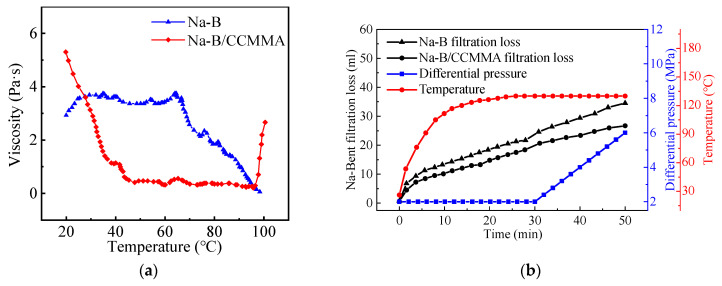
Influence of temperature on the performance of each system. (**a**) Rheological performance; (**b**) filtration performance.

The above results demonstrate that the addition of CCMMA enhances the viscosity of Na-B dispersion and reduces filtrate loss after hot treatment. However, the rheological and filtration behavior of CCMMA under real-time temperature conditions is not fully understood. To investigate this, temperature scanning rheology was conducted on Na-B and Na-B/CCMMA dispersions at a shear rate of 2 s^−1^. In Figure 11a, it can be observed that the apparent viscosity of Na-B and Na-B/CCMMA dispersions exhibits different trends with temperature. Below 70 °C, the viscosity of Na-B dispersion remains relatively constant, but it gradually decreases as the temperature reaches 99 °C, approaching 0.1 Pa·s. On the other hand, the viscosity of Na-B/CCMMA dispersion is initially higher than that of Na-B dispersion at 20 °C. However, it progressively decreases between 20 °C and 45 °C before stabilizing around 0.9 Pa·s with further temperature increase. Notably, Na-B/CCMMA also displays thermal thickening behavior at approximately 97 °C, which is about 10 °C higher than its aqueous solution’s response temperature. This suggests that CCMMA can enhance the colloidal stability of Na-B in dispersions and maintain its viscoelastic properties under high temperature conditions. However, the thermal thickening temperature of CCMMA in this study is higher than the temperatures mentioned earlier, indicating the potential application of CCMMA in maintaining viscosity in water-based drilling fluids under specific high-temperature conditions. Furthermore, when considering the rheological experiment results at room temperature after hot treatment, CCMMA demonstrates the ability to increase viscosity at high temperatures and recover its original rheological characteristics upon cooling, displaying thermal reversibility in Na-B dispersion. This reversibility makes CCMMA valuable for recycling in the water-based drilling fluid circulation process.

Further investigation was conducted on the filtration loss of CCMMA under high-temperature conditions, as shown in Figure 11b. The results reveal that CCMMA effectively mitigates filtrate loss under high-temperature and high-pressure conditions throughout the testing process. In contrast, the filtration volume of Na-B/CCMMA dispersion increases less when the temperature reaches around 110 °C and remains relatively constant until around 130 °C. There is a significant difference in the amount of water loss between Na-B and Na-B/CCMMA dispersions, especially after 110 °C. Following cross-linking, the PMAOEM side chains of CCMMA become hydrophobic, resulting in an increased hydrophobicity of the filter cake and preventing water intrusion. As the pressure difference steadily increases (up to 130 °C), the filter cakes become more compact and hydrophobic, exhibiting greater ability to withstand high pressures. This observation is attributed to the strong affinity of CCMMA for bentonite flakes and the improvement in the hydrophilic/hydrophobic balance of PMAOEM side chains through the formation of numerous hydrogen bonds.

**Figure 12:**
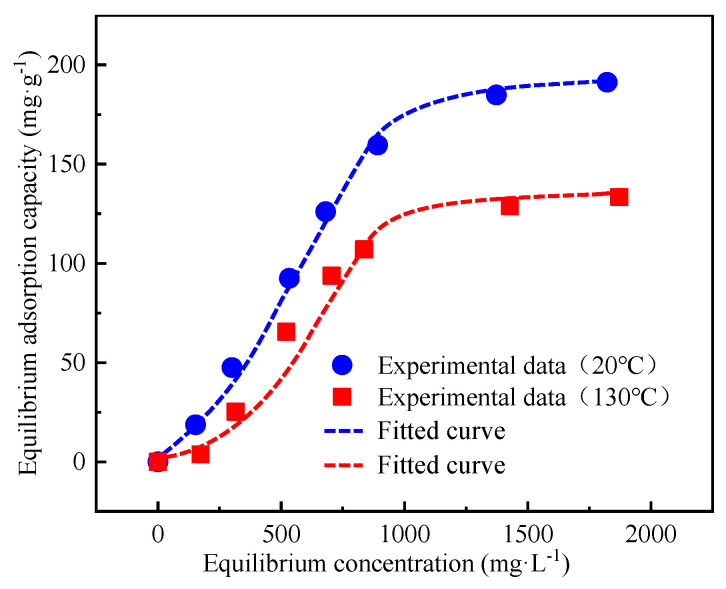
Curve of the Chapman sigmoidal equation.

Figure 12 and Table 3 present the adsorption isotherms of CCMMA on Na-B, which are comparable at temperatures of 20 °C and 130 °C. The Chapman sigmoidal equation provided the most accurate fit, suggesting that the adsorption of CCMMA on Na-B follows an S-shaped or reverse S-shaped isotherm. This indicates the presence of synergistic adsorption mechanisms [49,50,51]. At 20 °C under S-shaped isotherm conditions, the adsorption of CCMMA is minimal at low concentrations but increases significantly as the concentration increases due to various binding mechanisms such as intercalation, ionic bonding, and hydrogen bonding. At concentrations above 1400 mg/L, the adsorption capacity reaches a plateau, indicating saturation. At 130 °C, the adsorption behavior of CCMMA on Na-B is similar but with a reduced maximum adsorption capacity and lower fitting values, indicating partial desorption of CCMMA at temperatures above LCST. However, at low concentrations (<650 mg/L), the adsorption capacities of CCMMA are highly comparable, indicating a dominant intercalation binding mode. Multilayer adsorption occurs at high concentrations. Furthermore, the hydrophobic PMAOEM transformed at 130 °C enhances surface hydrophobic association and maintains a comparable adsorption capacity. Therefore, under high-temperature conditions (>LCST), the adsorption capacity of CCMMA is not weakened and it retains strong hydrophobic properties.

**Figure 13:**
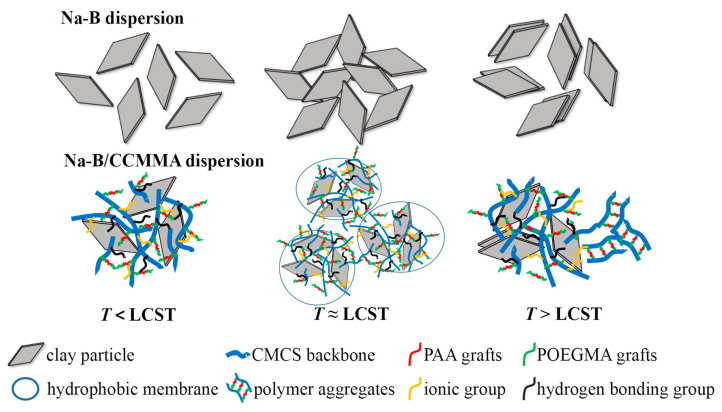
Schematic diagram of the interaction between Na-B and CCMMA in aqueous solution as temperature increases.

Figure 13 illustrates the interaction between bentonite flakes and CCMMA. As the temperature increases, the Na-B dispersion undergoes a transition from dispersion to flocculation and then to aggregation, resulting in a significant decrease in viscosity while keeping it almost unchanged initially. Additionally, the Na-B dispersion experiences a substantial fluid loss due to the inability to form a water-impermeable filter cake on the surface accumulation. The addition of CCMMA forms a network structure by hydrogen bonding and ionic bonding with bentonite, leading to an increase in initial viscosity. Additionally, the CCMMA-coated bentonite aggregates help reduce filtration loss. However, as the temperature rises, there is desorption of CCMMA, resulting in a loss of viscosity. When the temperature exceeds the LCST, the hydrophilicity of PMAOEM side chains changes. The resulting structure provides hydration and entropy stabilization effects for bentonite, preventing aggregation and promoting viscosity improvement. The hydrophobic interactions between particles form a barrier, reducing the filtrate loss of bentonite at high temperatures. In addition, partially desorbed PMAOEM side chains easily form deformable hydrophobic polymer aggregates (Na-B/CCMMA) at high temperatures. From the rheological results, these polymer aggregates have some resistance to shear stress, and they can also block the filter cake to reduce filtration loss.

### 3.5. Intelligent Plugging Performance

**Figure 14:**
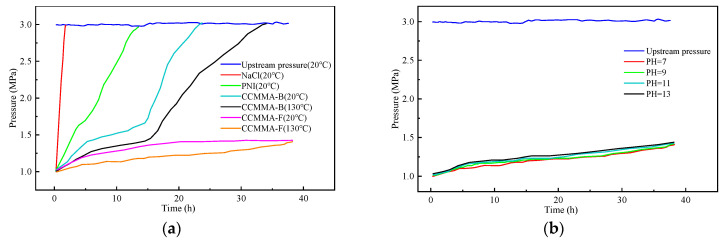
Results of pressure transmission experiment. (**a**) Effect of temperature; (**b**) effect of pH.

Figure 14a demonstrates that when the 3% NaCl solution is below its temperature response point, the pressure rapidly reaches equilibrium. This occurs because the NaCl solution does not impede the material, allowing the fluid to flow directly through the cracks and pores within the shale core. When tested with 3% PNI, there was a significant decrease in permeability concurrent with a significant increase in pressure. This suggests that PNI possesses a certain degree of plugging ability. When the blocking agent was a 3% mass fraction of CCMMA-B, the rate of pressure rise slowed down until it reached 1.68 MPa. However, the pressure rapidly increased due to a portion of CCMMA particles not effectively sealing the rock core. While providing a temporary sealing effect, the pressure gradually rose and eventually reached downstream values. After 16 h, the downstream pressure equilibrated with the upstream pressure. When compared to CCMMA-B, it was observed that using PNI as the plugging material resulted in a slower pressure rise and lower permeability after plugging.

Figure 14b reveals that the pH of the drilling fluid is alkaline, which enhances the sealing performance with a pH range of 7–13. The results indicate that pH has a minimal impact on sealing performance.

As a result, CCMMA demonstrates superior sealing effectiveness compared to PNI. When the temperature exceeded the response temperature, the permeability after using CCMMA-F as the blocking agent reduced significantly to 0.0295 × 10^−7^ μm^2^. Furthermore, after 30 h, the downstream pressure increased to 1.47 MPa and remained unchanged. Therefore, CCMMA can block the pores of the rock core more densely, which can fully reduce the intrusion of free water.

**Figure 15:**
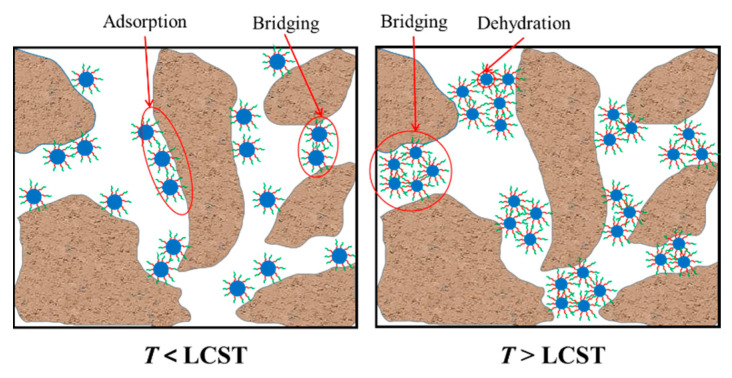
Schematic diagram of the plugging mechanism of CCMMA in micropores.

Figure 15 illustrates the plugging mechanism of CCMMA in micropore formation. Below the response temperature, the plugging action of CCMMA is related to its chemical structure and compatibility with the pore structure. When above the response temperature, all CCMMA enhances plugging ability, involving two processes for effective plugging. The first process is dehydration of CCMMA nanoassemblies within the core, enabling them to enter small-sized pore throats. Then, they fill larger pore spaces through interparticle hydrophobic interactions. Thermally induced aggregates possess hydrophobic properties, significantly closing off flow channels with increasing differential pressure driving elastic aggregates into narrower pore throats, enhancing plug strength. Therefore, the polymer after response exhibits good plugging effectiveness for pores of various sizes. Additionally, CCMMA undergoes partial disassembly below the response temperature, exhibiting responsive intelligent disassembly behavior due to its reversible LCST behavior.

## 4. Conclusions

This study successfully synthesized a novel microporous formation plugging agent called CCMMA and analyzed its unique characteristics. The results confirmed that CCMMA has the ability to self-assemble into nanomicelles via a combination of hydrophobic interactions between the furan-glucose ring, fatty chains, and methacrylate ester groups, as well as molecular hydrogen bonding between carboxyl, hydroxyl, and amino groups. These interactions contribute to the formation of a stable micelle core with MAOEM-co-AA serving as the hydrophilic shell of the graft.

Rheology and filtration property experiments revealed that CCMMA’s viscosity increases significantly with an increase in its content, resulting in a corresponding decrease in filtration loss. This is due to the transition from hydrophilicity to hydrophobicity, which endows CCMMA with excellent HTHP filtration reduction capacity and adsorption ability. Furthermore, Na-B/CCMMA dispersions demonstrate temperature-reversible viscosity properties as the viscosity can be restored after treatment at 130 °C.

Core plugging tests demonstrate that above a certain transition temperature, CCMMA exhibits remarkable plugging performance in low-permeability sandstone and medium-permeability carbonate rocks. This is primarily attributed to CCMMA’s temperature-responsive behavior and ability to rapidly respond to temperature changes. The plugging process is reversible, allowing for dynamic control of fluid loss in drilling operations.

This research achievement represents a significant advancement in improving the sealing effect of drilling fluid in formations with permeability leakage. By capitalizing on CCMMA’s intelligent reversible performance, drilling construction costs can be reduced while maintaining efficient and reliable operations. This innovative approach has the potential to transform the oil and gas exploration industry, providing a more sustainable and cost-effective solution for meeting escalating demand in microporous reservoirs.

## Figures and Tables

**Table 1:** Components of different CCMMA types.

	CMCS (g)	MAOEM (g)	AA (g)	MAOEM/AA (mol/mol)	APS (g)	GR (%)	GE (%)
CCMMA-A	1.20	6.00	0.00	10/0	0.06	360.67	72.11
CCMMA-B	1.20	5.90	0.10	9/1	0.06	380.46	76.09
CCMMA-C	1.20	5.78	0.22	8/2	0.06	390.46	78.13
CCMMA-D	1.20	5.63	0.37	7/3	0.06	401.27	80.27
CCMMA-E	1.20	5.45	0.55	6/4	0.06	398.00	79.56
CCMMA-F	1.20	5.21	0.79	5/5	0.06	419.22	83.84

**Table 2:** Effects of CCMMA on the performance of Na-B dispersions.

Test Samples	Test Conditions	YP (Pa)	PV (mPa·s)	AV (mPa·s)	Filtration Loss (mL)
Na-B dispersion	BHR	6.51	2.79	3.72	22.79
	AHR at 100 °C	13.49	9.30	4.19	27.53
	AHR at 115 °C	14.88	10.23	4.65	27.16
	AHR at 130 °C	6.74	6.05	0.70	26.78
Na-B dispersion + 2 wt% CCMMA	BHR	14.88	13.02	1.86	22.13
	AHR at 100 °C	12.56	10.23	2.33	25.48
	AHR at 115 °C	10.70	9.30	1.40	75.52
	AHR at 130 °C	14.42	12.09	2.33	14.51
Na-B dispersion + 4 wt% CCMMA	BHR	13.25	10.70	2.56	18.60
	AHR at 100 °C	10.23	8.37	1.86	58.59
	AHR at 115 °C	19.53	17.67	1.86	8.93
	AHR at 130 °C	17.21	14.88	2.33	13.21
Na-B dispersion + 6 wt% CCMMA	BHR	11.63	9.30	2.33	32.74
	AHR at 100 °C	6.51	4.65	1.86	13.95
	AHR at 115 °C	6.98	5.58	1.40	15.81
	AHR at 130 °C	5.58	5.12	0.47	17.67
Na-B dispersion + 2 wt% CMCS	BHR	6.51	2.79	3.72	22.79
	AHR at 100 °C	13.49	9.30	4.19	27.53
	AHR at 115 °C	14.88	10.23	4.65	27.16
	AHR at 130 °C	6.74	6.05	0.70	26.78

**Table 3:** Fitting parameters of three isotherm equations.

Fitting Equation	Fitting Parameters	T = 20 °C	T = 130 °C
Langmuir	q_m_ (mg/g)	338.32197	240.003535
	K_L_ (L/mg)	0.00063	0.000665
	R^2^	0.988365	0.873905
Freundlich	K_F_ (L/mg)	1.14471	1.21334
	n	1.557045	1.502995
	R^2^	0.972195	0.85614
Chapman sigmoidal equation	a	177.421335	134.679505
	b	0.002625	0.002945
	c	2.66448	3.0001
	R^2^	0.9994	0.949335

## Data Availability

Data are contained within the article.
